# Epidemiology of and Risk Factors for Amebic Meningoencephalitis, Kerala, India, 2025 

**DOI:** 10.3201/eid3209.260492

**Published:** 2026-09

**Authors:** Harikumar Sivanandan, Jeromie Wesley Vivian Thangaraj, Ala Saritha, Anitha Bhaskar, Aravind Reghukumar, Asuma A. Rahim, Bindu Vadakkayil, Chintha Sujatha, Karthika Meenakshy Amma, Manoj Arayakandy Thali, Prathibha M. Thankamoni Amma, Priya Chandran, Rekha Melathuparambil Ravindran, Remya Prakash, Sabitha Rose Jacob, Shameer Vadekkandiyil, Shubin Chenayil, Unnikrishnan Bhaskarannair, Abhishek Gopalakrishnan Saraswthy, Anitha Puduvail Moorkoth, Bhagyalakshmi Mahavan Kanchana, Biju George, Dinesh Gopal, Heera Hassan, Jincy Johnson, Kiran Raj Divakaran, M.C. Sathya Bhama, Megha Shaju, Minu Abraham, Neetha T. Ramankutty, Pressy Sankar, Priyanka Sajeev, Saritha Narayanankutty, O. Sasikumari, Sujina Cheraparambil Muthukutty, Thekkumkara Surendran Anish, Vishnunath Kadavath, Aruna Raman, Bindu Gopalakrishnan Santhakumari, Gayathri Ramakrishnan, Geetha Panarkandy, Jayakrishnan Machinery Puthanpurayil, Mohandas Nair Karippoth, Shameem Anathan Mohammed, Sheeja Sugunan, Sindhu Thekkile Gangadharan, Anuja Ushakumari, Chandni Radhakrishnan, Easwaran Sreekumar, Naveen Anaswara, Pillaveetil Sathyadas Indu, Reetha Kakkarakandiyil Pareri, Sahadevan Sunija, Sajeeth Kumar Keeriyatt Govindan, Jaichand Johnson, Ajan Maheswaran Jaya, Abey Sushan, Raman Swathy Vaman, Lakshmi Geetha Gopalakrishnan, Arun P. Vijayalekshmi, Dinesh Kumar Harikrishnan, Sabarinathan Ramasamy, Navaneeth S. Krishna, Viswanathan V. Kollengode, Reena K. Joseph, Vinay Goyal, Rajan Khobragade, Manoj V. Murhekar, AME Study Group

**Affiliations:** Government of Kerala Directorate of Health Services, Thiruvananthapuram, India (H. Sivanandan, M.A. Thali, R.M. Ravindran, S. Chenayil, U. Bhaskarannair, A.G. Saraswthy, K.R. Divakaran, M. Abraham, P. Sajeev, R.K. Pareri, A.M. Jaya, A. Sushan, R. Vaman, L.G. Gopalakrishnan, A.P. Vijayalekshmi, R.K. Joseph); Indian Council of Medical Research National Institute of Epidemiology, Chennai, India (J.W.V. Thangraj, A. Saritha, D. Gopal, M. Shaju, V. Kadavath, D.K. Harikrishnan, S. Ramasamy, N.S. Krishna, M.V. Murhekar); Government Medical College, Thiruvananthapuram (A. Bhaskar, A. Reghukumar, K.M. Amma, R. Prakash, B.M. Kanchana, H. Hassan, J. Johnson, M.C.S. Bhama, N.T. Ramankutty, S. Narayanankutty, O. Sasikumari, A. Raman, B.G. Santhakumari, S. Sugunan); Government Medical College, Kozhikode, India (A.A. Rahim, B. Vadakkayil, S. Vadekkandiyil, A.P. Moorkoth, P. Sankar, G. Ramakrishnan, G. Panarkandy, J.M. Puthanpurayil, M.N. Karippoth, S.A. Mohammed, S.K.K. Govindan); Government Medical College, Kollam, India (C. Sujatha, P.M. Thankamoni Amma); Government Medical College, Manjeri, India (P. Chandran, S.R. Jacob, B. George, S.C. Muthukutty); Kerala One Health Centre for Nipah Research and Resilience, Kozhikode (T.S. Anish); Government Medical College, Kasaragod, India (S.T. Gangadharan); Directorate of Medical Education, Kerala, India (A. Ushakumar, V.V. Kollengode); Government Medical College, Wayanad, India (C. Radhakrishnan); Institute of Advanced Virology, Thiruvananthapuram (E. Sreekumar); Kerala Centre for Disease Control and Prevention, Thiruvananthapuram (N. Anaswara); Government Medical College, Thrissur, India (P.S. Indu); State Public Health and Clinical Lab, Thiruvananthapuram (S. Sahadevan); National Health Mission, Government of Kerala, Thiruvananthapuram (V. Goyal); Health & Family Welfare, Government of Kerala, Thiruvananthapuram (R. Khobragade)

**Keywords:** amebic meningoencephalitis, meningitis/encephalitis, central nervous system, protozoal infections, Naegleria fowleri, Acanthamoeba, risk factors, parasites, waterborne illness, India

## Abstract

Amebic meningoencephalitis (AME) is a highly fatal central nervous system infection caused by various free-living amebae. We describe epidemiology of AME cases from Kerala, India, during January 1–November 11, 2025, and identify behavioral risk factors associated with real-time PCR-confirmed *Acanthamoeba* spp. infection through a matched case–control study. We identified 159 AME case-patients, of which 67 (42.1%) tested positive for *Acanthamoeba* spp. amebae. Most AME cases were reported from 4 districts, peaking during the monsoon and postmonsoon periods. Exposure to natural water bodies for swimming or bathing and using nonchlorinated water for domestic purposes were associated with AME. History of maxillofacial injury or nasal surgery with subsequent exposure to natural water bodies and washing ulcers or skin lesions in natural water bodies were associated with higher odds of disease. Targeted risk communication about safe water practices and routine chlorination of domestic water sources could help reduce AME risk in affected communities.

Amebic meningoencephalitis (AME) is a collection of rare but frequently fatal infections of the central nervous system caused by various free-living amebae (FLA). Such infections include primary amebic meningoencephalitis (PAM), caused by *Naegleria fowleri* amebae, and granulomatous amoebic encephalitis (GAE), caused by different species of *Acanthamoeba*, *Balamuthia mandrillaris*, and *Sappinia* spp. amebae (*1*,*2*). PAM typically manifests as an acute, rapidly progressive meningoencephalitis, whereas GAE usually manifests as a subacute or chronic encephalitic illness. The clinical manifestations of PAM are indistinguishable from those of bacterial meningitis, whereas GAE can mimic brain abscess, encephalitis, or meningitis. PAM and GAE are associated with high mortality rates; case-fatality rates >90% have been reported globally (*3*,*4*).

*N. fowleri* amebae infect susceptible persons when contaminated water enters the nasal cavity; trophozoites can penetrate the olfactory mucosa, cross the cribriform plate, and invade the olfactory bulb, leading to parenchymal inflammation and tissue damage (*5*,*6*). The trophozoites of *Acanthamoeba* spp. amebae can enter the body through the eyes, nasal passages, or ulcerated or broken skin (*7*,*8*). Ocular exposure can cause severe keratitis (*9*); entry through the respiratory tract or broken skin can cause hematogenous spread to the central nervous system (*10*,*11*). Swimming or diving in warm fresh water like hot springs, lakes, rivers, and ponds are the primary risk factors for *N. fowleri* infection, although other water sources, including tap water, have been implicated (*12*). Environmental risk factors for *Acanthamoeba* GAE include exposure to contaminated soil, dust, freshwater sources, and untreated water systems. Because *Acanthamoeba* is ubiquitous in natural and manmade environments, inhalation of airborne cysts or contamination of skin lesions can result in infection, particularly in susceptible persons (*13*–*15*).

In the state of Kerala, India, 45 cases of AME were reported during 2016–2024, including 15 deaths (33% case-fatality rate). *N. fowleri* accounted for 5 of 7 cases reported during 2016–2023, whereas 38 cases and 8 deaths were reported in 2024, most of which were caused by *Acanthamoeba.* In response to the increasing number of AME cases, the Department of Health and Family Welfare, Government of Kerala, formulated a multipronged action plan in 2025 to improve the diagnosis and management of AME cases (*16*).

Descriptive epidemiology of AME in Kerala has not been documented in published literature. In this context, we aimed to describe the AME cases reported in 2025 in Kerala by time, place, person, and laboratory diagnosis. In addition, we conducted a case–control study to identify behavioral risk factors associated with real-time PCR (rPCR)–confirmed cases of amebic meningoencephalitis caused by *Acanthamoeba* spp. amebae.

## Methods

### Descriptive Epidemiology

We defined a case as a patient experiencing clinical features suggestive of acute meningoencephalitis, including fever, headache, vomiting, neck stiffness, altered sensorium, seizures, or other neurologic symptoms, diagnosed during January 1–November 11, 2025, and with laboratory evidence of infection with FLA. We defined laboratory evidence as either demonstration of motile trophozoites in cerebrospinal fluid (CSF) by wet-mount microscopy or detection of FLA DNA in CSF by rPCR targeting species-specific 18S rRNA genes for *N. fowleri*, *Acanthamoeba* spp., *Balamuthia mandrillaris*, *Paravahlkampfia* spp., or *Vermamoeba vermiformis.* Molecular assays included conventional pangenus FLA and genus-specific PCR for *Acanthamoeba*, *Naegleria*, and *Balamuthia* and rPCR (*17*,*18*) (Appendix).

#### Case Search

We identified cases among patients with suspected AME admitted to the Government Medical College, Thiruvananthapuram and Kozhikode, India. We collected information on demographic characteristics, area of residence, clinical manifestation, underlying conditions, date of admission, and outcome status from patients, family members, and hospital admission records.

#### Data Analysis

We plotted the geographic locations of cases and constructed an epidemic curve to describe the spatial and temporal distribution of cases. We summarized age using median and interquartile range (IQR) and categorical variables using frequencies and percentages. We calculated median durations from date of illness onset to admission and from admission to death. We analyzed all characteristics separately for rPCR-confirmed *Acanthamoeba* spp. cases, rPCR-confirmed *N. fowleri* cases, and cases diagnosed by wet-mount microscopy.

### Analytical Epidemiology

We conducted a matched case–control study in the Kollam, Kozhikode, Malappuram, and Thiruvananthapuram districts of Kerala during October–November 2025. We defined a case-patient as a resident of Kollam, Kozhikode, Malappuram, or Thiruvananthapuram district who experienced clinical features suggestive of AME and received a diagnosis during July–November 2025 that was confirmed by detection of FLA DNA for *Acanthamoeba* spp. in CSF by rPCR. We restricted the analytical study to rPCR-confirmed *Acanthamoeba* spp. cases because wet-mount microscopy does not identify the amebic species and because transmission pathways and risk factors may differ by species. Controls were apparently healthy persons without a history of AME or meningoencephalitis, matched to case-patients by age (+3 years), sex, and place of residence. We sought to include all cases diagnosed during July–November in the study.

#### Selection of Cases and Controls

The study hospitals prepared a linelist of patients with rPCR-confirmed *Acanthamoeba* spp. AME diagnosed during July–November 2025 residing in the 4 study districts. Trained field investigators visited each case household to obtain consent and collect data. For each case-patient, we prepared a list, with the assistance of a local health worker, of apparently healthy persons who had no history of AME or meningoencephalitis and met the matching criteria. We randomly selected 3 controls from this list for each case-patient.

#### Human Participant Protection

Institutional Human Ethics Committee of the Indian Council of Medical Research—National Institute of Epidemiology and participating institutions approved the study protocol (NIE/IHEC/202509-01). We obtained written informed consent from adult participants >18 years of age and from the parents or guardians of minors <18 years of age. We acquired assent from children 7–17 years of age.

#### Data Collection

We collected data during October–November 2025. Trained investigators interviewed case-patients and controls at their homes using a structured questionnaire created with Open Data Kit (https://getodk.org). The questionnaire collected information on demographic and socioeconomic characteristics, water sources used for domestic purposes, water-related activities, nasal rinsing practices, chlorination of well water, and underlying conditions suggestive of immunocompromised status, including diabetes, chronic kidney disease, malignancy, or HIV infection. We defined domestic water use as water used for activities involving direct contact with the face, head, nose, or body, such as bathing or washing the face or hair; it did not include washing clothes or utensils. For case-patients, we assessed exposures during the 3 months before illness onset. For controls, we assessed exposures during the corresponding 3-month reference period before illness onset of the matched case-patient. Water-related activities included swimming or bathing in ponds, lakes, rivers, canals, streams, or swimming pools; entry into natural water bodies; domestic or occupational contact with pond, river, canal, stream, or well water other than swimming; nasal irrigation; and nasal ablution as part of ritual cleansing. For case-patients who died, we obtained information from a reliable family member.

### Statistical Analysis

We summarized sociodemographic characteristics of cases and controls using frequencies and percentages. We performed univariable conditional logistic regression analysis to estimate the unadjusted matched odds ratios (MOR) along with 95% CIs. We used multivariable conditional logistic regression to estimate adjusted matched odds ratios (aMOR) and 95% CIs. We constructed directed acyclic graphs using DAGitty software (http://www.dagitty.net) to represent hypothesized causal pathways between exposures and AME (Appendix Figure 1). We estimated population attributable fractions (PAF) along with 95% CIs for selected modifiable exposures. We applied the backdoor criterion to identify the minimal sufficient adjustment set for each risk factor. We performed all statistical data analysis using Stata version 17 (StataCorp LLC, https://www.stata.com).

## Results

### Descriptive Epidemiology

We identified 159 AME case-patients during January 1–November 11, 2025. Of those, 76 (47.8%) were confirmed by rPCR and 83 (52.2%) by wet-mount microscopy. Among the 76 PCR-confirmed case-patients, 67 (88.2%) were positive for *Acanthamoeba* spp. and 9 (11.8%) for *N. fowleri* amebae. The median age was 43 (IQR 29–58) years among rPCR-confirmed *Acanthamoeba* spp. case-patients, 34 (IQR 18–38) years among rPCR-confirmed *N. fowleri* case-patients, and 39 (IQR 14–55) years among case-patients who tested positive by wet-mount microscopy. Male patients accounted for 58.2% and female patients 41.8% of rPCR-confirmed *Acanthamoeba* spp. case-patients; 60.2% of case-patients positive by wet microscopy were male and 39.8% female (Table 1).

**Table 1:** Demographic distribution and clinical characteristics of all case-patients in study of amebic meningoencephalitis, Kerala state, India, 2025*

Characteristic	rPCR		CSF wet-mount microscopy, n = 83	Total, N = 159
	*Acanthamoeba*, n = 67	*Naegleria*, n = 9		
Sex				
F	28 (41.8)	5 (55.6)	33 (39.8)	66 (41.5)
M	39 (58.2)	4 (44.4)	50 (60.2)	93 (58.5)
Age, y				
0–5	2 (3.0)	1 (11.1)	2 (2.4)	5 (3.1)
6–17	9 (13.4)	1 (11.1)	26 (31.3)	36 (22.7)
18–45	27 (40.3)	5 (55.6)	24 (28.9)	56 (35.2)
>45	29 (43.3)	2 (22.2)	31 (37.4)	62 (39.0)
Mean (+SD)	41.9 (20.7)	32.1 (20.4)	36.3 (21.7)	38.4 (21.3)
Median (IQR)	43 (29–58)	34 (18–38)	39 (14–55)	40 (17–56)
Underlying condition				
Y	28 (41.8)	3 (33.3)	31 (37.3)	62 (39.0)
N	39 (58.2)	6 (66.7)	52 (62.7)	97 (61.0)
Underlying condition suggestive of immunocompromise				
Y	15 (22.4)	3 (33.3)	23 (27.7)	41 (25.8)
N	52 (77.6)	6 (66.7)	60 (72.3)	118 (74.2)
Clinical outcome				
Died	17 (25.4)	4 (44.4)	20 (24.1)	41 (25.8)
Survived	50 (74.6)	5 (55.6)	63 (75.9)	118 (74.2)
Days from hospital admission to death	n = 17	n = 4	n = 20	n = 41
Median (IQR)	34 (25–45)	28 (19.5–30)	27.5 (14.2–37)	31 (19−42)
Days from illness onset to hospital admission	n = 52	n = 9	n = 64	n = 125
Median (IQR)	3 (0–13)	4 (2–26)	4 (1–11)	4 (1–13.5)
Clinical manifestations	n = 64	n = 8	n = 76	n = 148
Fever	32 (50.0)	6 (75.0)	48 (63.2)	86 (58.1)
Headache	34 (53.1)	5 (62.5)	44 (57.9)	83 (56.1)
Vomiting	18 (28.1)	5 (62.5)	31 (40.8)	54 (36.5)
Seizures	11 (17.2)	4 (50.0)	14 (18.4)	29 (19.6)
Altered sensorium	11 (17.2)	1 (12.5)	15 (19.7)	27 (18.2)
Weakness in the upper limb/lower limb	13 (20.3)	3 (37.5)	8 (10.5)	24 (16.2)
Neck stiffness/meningeal signs	5 (7.8)	1 (12.5)	15 (19.7)	21 (14.2)
Myalgia/fatigue	7 (10.9)	0	13 (17.1)	20 (13.5)
Diplopia	6 (9.4)	0	5 (6.6)	11 (7.4)
Blurred vision	6 (9.4)	0	3 (3.9)	9 (6.1)
Dizziness/vertigo	6 (9.4)	1 (12.5)	2 (2.6)	9 (6.1)
Loss of consciousness	4 (6.3)	0	5 (6.6)	9 (6.1)
Slurred speech	4 (6.3)	1 (12.5)	3 (3.9)	8 (5.4)
Sensory disturbance	7 (10.9)	0	0	7 (4.7)
Chills/rigors	3 (4.7)	0	2 (2.6)	5 (3.4)
Photophobia	3 (4.7)	0	2 (2.6)	5 (3.4)
6th cranial nerve palsy/upper motor neuron/ lower motor neuron facial palsy	1 (1.6)	0	2 (2.6)	3 (2.0)
Ptosis	2 (3.1)	0	0	2 (1.4)
Involuntary movement	2 (3.1)	0	0	2 (1.4)

*Values are no. (%) except as indicated. CSF, cerebrospinal fluid; IQR, interquartile range; rPCR, real-time PCR.

Among the 159 case-patients, 41 (25.8%) died. Seventeen (25.4%) deaths were among rPCR-confirmed *Acanthamoeba* spp. case-patients, 4 (44.4%) were among rPCR-confirmed *N. fowleri* case-patients, and 20 (24.1%) were among wet-mount microscopy case-patients. The median duration from illness onset to hospitalization was 4 (IQR 1–13.5) days and broadly similar across the 3 groups. Among the case-patients who died, the median duration from hospital admission to death was 31 (IQR 19–42) days. Overall, 62 (39.0%) case-patients had >1 underlying condition. Among rPCR-confirmed *Acanthamoeba* spp. case-patients, 15 (22.4%) had a condition suggestive of an immunocompromising condition or a history of immunosuppressive therapy (Table 1).

**Figure 1:**
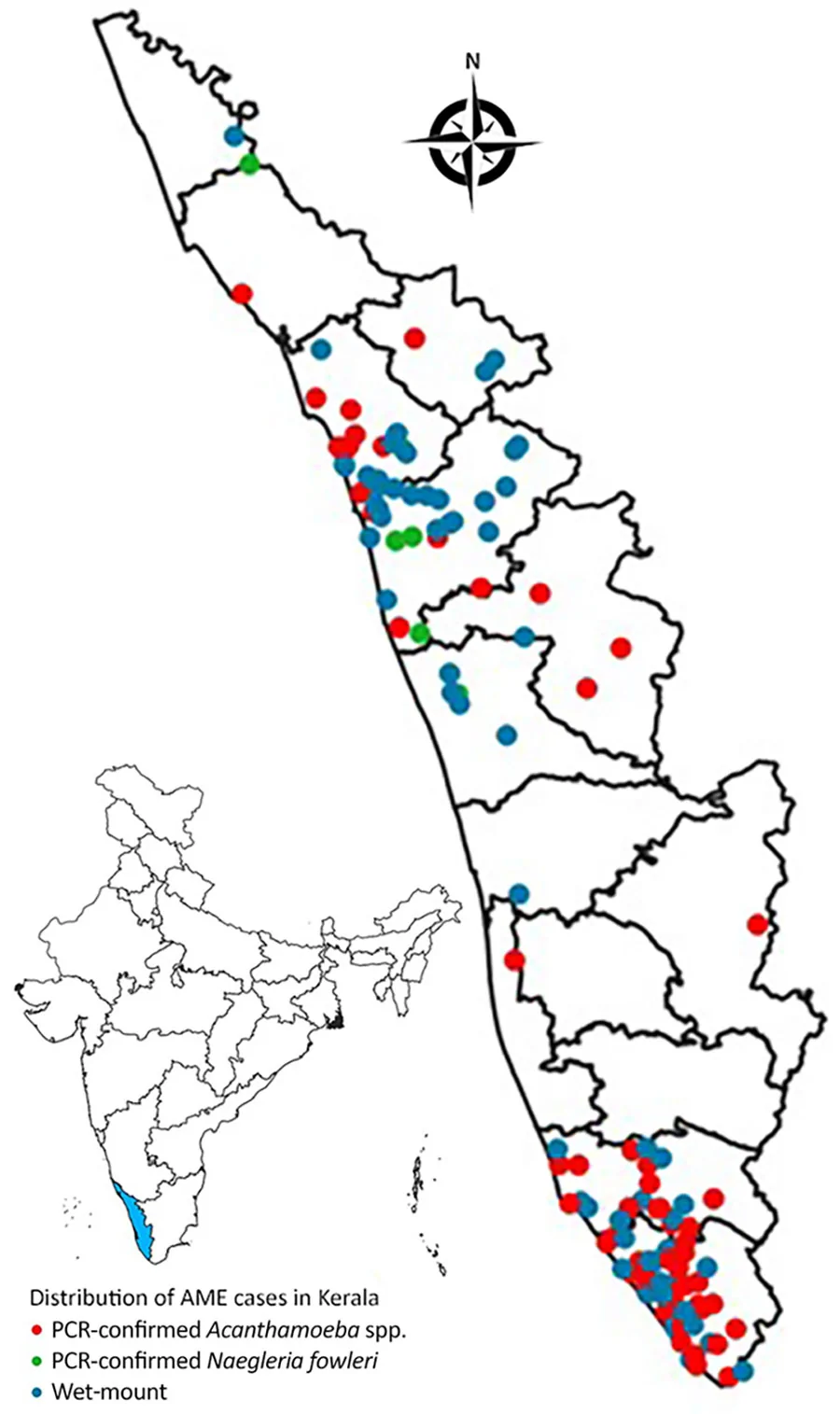
Locations of reported cases of amebic meningoencephalitis in Kerala state, India, 2025. Cases were confirmed by real-time PCR as caused by *Acanthamoeba* spp. or *Naegleria fowleri* amebae or confirmed via wet-mount microscopy of cerebrospinal fluid. Inset map shows location of Kerala in India.

**Figure 2:**
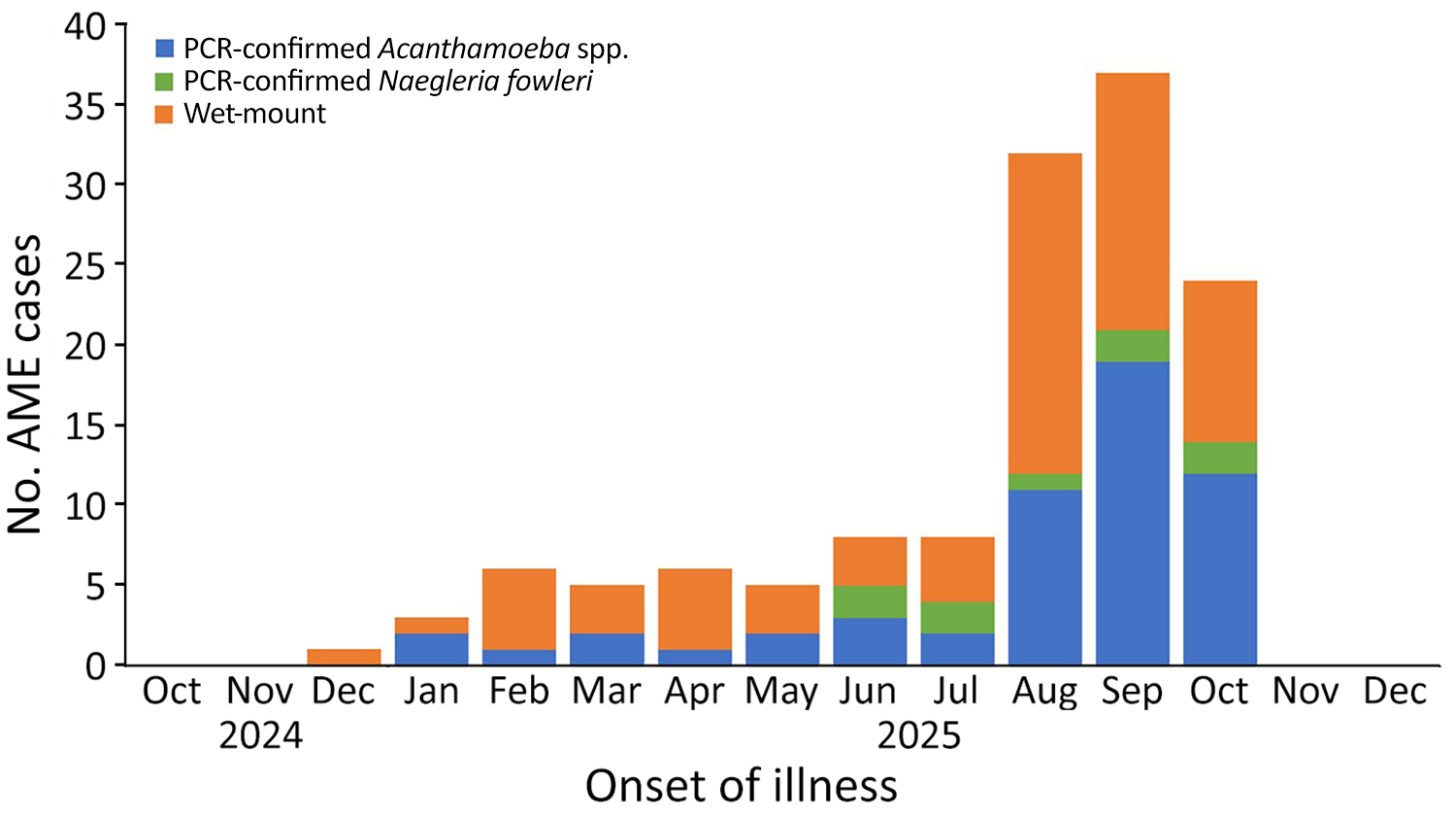
Epidemic curve of AME cases by onset of illness, Kerala state, India, 2025. Cases were confirmed by real-time PCR as caused by *Acanthamoeba* spp. or *Naegleria fowleri* amebae or confirmed via wet-mount microscopy of cerebrospinal fluid. The case shown in December 2024 was not confirmed until 2025. AME, amebic meningoencephalitis.

Most AME cases were reported from Thiruvanathapuram, Kollam, Kozhikode, and Malappuram districts (Figure 1). The epidemic curve showed a peak in cases during August–October, corresponding to the monsoon and postmonsoon period in Kerala (Figure 2).

The most common clinical signs and symptoms were fever (n = 86 [58.1%]), headache (n = 83 [56.1%]), vomiting (n = 54 [36.5%]), seizures (n = 29 [19.6%]), altered sensorium (n = 27 [18.2%]), neck stiffness (n = 21 [14.2%]), and loss of consciousness (n = 9 [6.1%]). Some patients also experienced less typical symptoms, such as diplopia, blurred vision, sensory disturbances, and cranial nerve palsies (Table 1). A total of 103 (81.7%) patients had CSF pleocytosis; 90 (70.3%) had lymphocytic predominance (Appendix Table 1).

### Analytic Epidemiology

During July–November 2025, we identified a total of 46 rPCR-positive *Acanthamoeba* spp. AME case-patients from the 4 study districts. We enrolled 43 of those case-patients and 129 controls in the study. Among the 43 case-patients, 5 (11.6%) were <18 years of age and 23 (53.5%) were >45 years of age; 24 (55.8%) were male and 19 (44.2%) female. Most case-patients resided in rural areas (n = 35 [81.4%]). Almost all participants (97.7% case-patients and 100% controls) lived in pucca or semi-pucca houses. A higher percentage of case-patients (74.4%) than controls (48.8%) belonged to a low socioeconomic group, as indicated by yellow or pink ration card status (Table 2).

**Table 2:** Sociodemographic profile of rPCR-confirmed *Acanthamoeba* spp. case-patients and controls in study of amebic meningoencephalitis, Kerala state, India, 2025*

Characteristic	Case-patients, n = 43	Controls, n = 129	p value
Age group, y			0.581
<5	0	4 (3.1)	
6–10	4 (9.3)	5 (3.9)	
11–17	1 (2.3)	6 (4.7)	
18–45	15 (34.9)	47 (36.4)	
>45	23 (53.5)	67 (51.9)	
Mean age (+SD)	46.2 (3.1)	46.1 (1.8)	
Sex			
M	24 (55.8)	72 (55.8)	NA
F	19 (44.2)	57 (44.2)	
District			
Kollam	12 (27.9)	36 (27.9)	NA
Kozhikode	7 (16.3)	21 (16.3)	
Malappuram	3 (7.0)	9 (7.0)	
Trivandrum	21 (48.8)	63 (48.8)	
Residence			NA
Rural	35 (81.4)	105 (81.4)	
Urban	8 (18.6)	24 (18.6)	
Type of house			1.000
Pucca/semi-pucca	42 (97.7)	129 (100)	
Kutcha	1 (2.3)	0	
Color of ration card			0.007
Yellow/pink	32 (74.4)	63 (48.8)	
Others	11 (25.6)	66 (51.2)	
Education level			0.577
Degree/above	5 (11.6)	15 (11.6)	
High school/higher secondary	20 (46.5)	71 (55.0)	
Primary/middle	15 (34.9)	33 (25.6)	
Illiterate/read-write	2 (4.7)	4 (3.1)	
Not applicable	1 (2.3)	6 (4.7)	

*Values are no. (%) except as indicated. NA, not applicable because cases and controls were matched for this variable; rPCR, real-time PCR.

In univariable conditional logistic regression analysis, case-patients had higher odds than controls of belonging to a low socioeconomic background (MOR = 2.8 [95% CI 1.3–5.9]) and using nonchlorinated water for domestic purposes, defined as water from unchlorinated household or public wells (MOR = 2.9 [95% CI 1.1–7.8]). Case-patients also had higher odds of exposure to natural water bodies, including ponds, lakes, rivers, canals, and streams, for swimming or bathing or entry into natural water bodies during the preceding 3 months (MOR = 3.6 [95% CI 1.6–8.5]) (Table 3).

**Table 3:** Risk factors associated with *Acanthamoeba*-caused cases by univariate conditional logistic regression analysis in study of amebic meningoencephalitis, Kerala state, India, 2025*

Risk factor	No. (%) cases, n = 43	No. (%) controls, n = 129	Matched odds ratio (95% CI)	p value
Occupation, painter/construction worker/farmer	5 (11.6)	13 (10.1)	1.2 (0.4–3.7)	0.768
Persons living below the poverty line	32 (74.4)	63 (48.8)	2.8 (1.3–5.9)	0.007
Use of nonchlorinated water for domestic use, household/public well, not chlorinated†	14 (50.0)	30 (32.6)	2.9 (1.1– 7.8)	0.029
Presence of fish in well†	1 (3.6)	6 (6.5)	2.9 (0.3– 31.5)	0.374
Cleaned well in previous 3 mo†	2 (7.1)	15 (16.3)	0.3 (0.0- 2.2)	0.223
Cleaned the overhead tank in previous 3 mo	16 (37.2)	60 (46.5)	0.7 (0.3–1.4)	0.275
Observed turbidity or a change in taste/smell in the water	10 (23.3)	21 (16.3)	1.7 (0.7–4.5)	0.253
Exposure to water bodies such as ponds/lakes/rivers/canals/streams for swimming or bathing or entered water body in previous 3 mo	18 (41.9)	25 (19.4)	3.6 (1.6–8.5)	0.003
History of swimming in swimming pool in previous 3 mo	1 (2.3)	0	NA	NA
Reached the bottom of lakes/ponds/canals/rivers/streams during swimming/bathing/diving or experienced Tharipu	8 (18.6)	7 (5.4)	3.7 (1.3–10.8)	0.015
Practised nasal rinsing in previous 3 mo	15 (34.9)	37 (28.7)	1.4 (0.6–3.2)	0.401
Smoking‡	8 (18.6)	21 (16.3)	1.2 (0.4–3.6)	0.686
Consumption of alcohol‡	9 (20.9)	34 (26.4)	0.6 (0.2–1.7)	0.349
Use of nasal snuff‡	0	4 (3.1)	NA	NA
Immunocompromised person§	15 (34.9)	33 (25.6)	1.9 (0.8–4.7)	0.173
Use of steroids in previous 3 mo	6 (13.9)	12 (9.3)	1.6 (0.6–4.6)	0.390
Experienced recurrent sinusitis in previous 3 mo	6 (13.9)	10 (7.8)	2.1 (0.7–6.7)	0.214
Had maxillofacial injury or nasal surgery and exposure to ponds/lakes/canals/rivers/streams	7 (16.3)	6 (4.7)	3.9 (1.2–12.4)	0.022
Hospitalization for severe COVID-19	2 (4.7)	11 (8.5)	0.5 (0.1–2.4)	0.392
Soil/dirt contact during farming/gardening in previous 3 mo	25 (58.1)	75 (58.1)	1 (0.5–2.1)	1.000
Cleaned the air conditioner in previous 3 mo	1 (2.3)	8 (6.2)	0.4 (0.0–2.9)	0.337
Used sprinklers/hose to water plants/wash vehicles in previous 3 mo	17 (39.5)	51 (39.5)	1 (0.5–2.1)	1.000
Had any ulcers/skin lesions in previous 3 mo	17 (39.5)	15 (11.6)	4.7 (2.1–10.8)	0.001
Washed ulcers/skin lesions in ponds/lakes/rivers/canals/streams	9 (20.9)	1 (0.8)	27 (3.4–213.1)	0.002

*NA, not applicable; Tharipu, water forcefully entering the nose. †Cases, n = 28; controls, n = 92. ‡Current or past. §Diabetes, HIV, chronic kidney disease, malignancy, or immunosuppressive therapy.

Other exposures more frequently reported among case-patients included reaching the bottom of a lake, pond, canal, river, or stream while swimming, bathing, or diving or experiencing Tharipu (a local term for water forcefully entering the nose). Case-patients were also more likely than controls to report a history of maxillofacial injury or nasal surgery followed by exposure to natural water bodies. A higher percentage of case-patients also reported washing ulcers or skin lesions in the natural water bodies (Table 3).

In multivariable conditional logistic regression analysis, exposure to natural water bodies for swimming, bathing, or any entry into such water bodies during the preceding 3 months was associated with AME (aMOR 4.7 [95% CI 1.8–12.1]; PAF 32.9% [95% CI 24.0%–40.8%]). Use of nonchlorinated water for domestic purposes was also independently associated with AME (aMOR 2.9 [95% CI 1.1–8.2]; PAF 34.2% [95% CI 13.5%–49.9%]). Case-patients had higher odds of having washed ulcers or skin lesions in the natural water bodies, although the CI was wide (aMOR 25.4 [95% CI 3.1–210.3; PAF 20.1% [95% CI 18.3%**–**21.8%]). A history of maxillofacial injury or nasal surgery followed by exposure to ponds, lakes, canals, rivers, or streams was also associated with AME (aMOR 4.8 [95% CI 1.3–16.7]; PAF 12.8% [95% CI 8.5%**–**17.1%]). Tharipu during swimming, bathing, or diving was associated with AME (aMOR 4.8 [95% CI 1.5–15.4]; PAF 14.7% [95% CI 10.1%–19.1%]) (Table 4).

**Table 4:** Risk factors associated with *Acanthamoeba*-caused cases by multivariable conditional logistic regression analysis in study of amebic meningoencephalitis, Kerala, India, 2025*

Risk factors	Univariate MOR (95% CI)	Adjusted MOR (95% CI)	PAF, % (95% CI)
Exposure to water bodies such as ponds/lakes/rivers/canals/streams for either swimming or bathing or entered a water body in previous 3 mo	3.6 (1.6–8.5)	4.7 (1.8–12.1)†	32.9 (24.0–40.8)
Use of nonchlorinated water for domestic use, household/public well, not chlorinated	2.9 (1.1–7.8)	2.9 (1.1–8.2)†	34.2 (13.5–49.9)
Washed ulcers/skin lesions in ponds/lakes/rivers/canals/streams	27 (3.4–213.1)	25.4 (3.1–210.3)†	20.1 (18.3–21.8)
Had a maxillofacial injury or undergone nasal surgery in the past, and exposure to ponds/lakes/canals/rivers/streams	3.9 (1.2–12.4)	4.8 (1.3–16.7)†	12.8 (8.5–17.1)
Reached the bottom of lakes/ponds/canals/rivers/streams during swimming/bathing/diving, or experienced Tharipu	3.7 (1.3–10.8)	4.8 (1.5–15.4)‡	14.7 (10.1–19.1)

*MOR, matched odds ratio; PAF, population attributable fraction; Tharipu, water forcefully entering the nose. †Adjusted for socioeconomic status ‡Adjusted for socioeconomic status and immunocompromised status.

## Discussion

In 2025, Kerala reported an increased number of laboratory-supported AME cases; *Acanthamoeba* spp. amebae accounted for most rPCR-confirmed cases. Case-patients were reported from several districts, peaking during monsoon and postmonsoon months. The substantial case-fatality rates among rPCR-confirmed cases underscored the severity of AME. *Acanthamoeba* GAE accounted for most rPCR-confirmed AME cases in Kerala during 2025. Increased clinical suspicion, wider availability of molecular diagnostics, and improved surveillance could have contributed to the higher number of detected cases; temporal clustering also suggested water-related environmental exposures.

In the matched case–control study restricted to rPCR-confirmed *Acanthamoeba* spp. cases, we identified several exposures associated with the illness. Those included washing ulcers or skin lesions in natural water bodies, exposure to natural water bodies among persons with history of maxillofacial injury or nasal surgery, use of nonchlorinated well water for domestic activities, exposure to natural water bodies for swimming or bathing, and Tharipu. Those findings suggest that both environmental exposures and potential portals of entry affect infection risk. The association between washing ulcers or skin lesions in natural water bodies and AME observed in our study is consistent with the known ability of *Acanthamoeba* spp. amebae to enter through breaks in the skin and subsequently disseminate to the CNS (*13*). Similarly, exposure to natural water bodies after maxillofacial injury or nasal surgery was associated with AME. Although the CIs around some of our estimates were wide, those associations are biologically plausible; such conditions could increase the opportunity for environmental organisms to enter tissue or the respiratory tract. Hence, public health communication should specifically advise persons with open wounds, chronic ulcers, or recent history of maxillofacial surgery to avoid exposure to natural water bodies. 

*Acanthamoeba* spp. amebae are widely distributed in the environment and have been isolated from soil, freshwater, wells, swimming pools, domestic water systems, and tap water (*1*,*19*). An environmental survey in 51 villages that reported cases found that, in 43 (84.3%) villages, >1 environmental water sources, including wells, ponds, drinking water sources, and water tanks, tested positive for FLAs (data not shown). Those environmental findings should be interpreted as supportive ecologic evidence only, because sampling was not designed to establish source attribution. 

For *N. fowleri* infection, entry of contaminated water through the nose during swimming and bathing, as well through Tharipu, is a definitive route of transmission (*12*). However, those are not well-established routes of transmission for *Acanthamoeba* GAE (*13*–*15*). In a case series of 173 nonkeratitis *Acanthamoeba* infections in the United States during 1956–2020, 10% had exposure to natural water bodies (*19*). Similarly, a review article from India identified 42 case reports, of which 2 had documented exposure to natural water bodies (*11*). A few case reports of *Acanthamoeba* spp. CNS infection among persons with near-drowning events have been documented (*20*,*21*). The associations between *Acanthamoeba* spp. AME and exposure to natural water bodies, use of nonchlorinated well water, and Tharipu observed in our study are epidemiologic associations and do not prove that water exposure led directly to neuroinvasion. Those findings might indicate contact with environmental sources where *Acanthamoeba* spp. amebae are present and possible entry into body by inhalation or respiratory route, water entering the nose during swimming or bathing, or other behaviors associated with untreated water use that we did not measure. Further environmental, clinical, and microbiological studies are needed to determine whether that association reflects a route of acquisition or an epidemiologic marker of exposure to contaminated water.

In Kerala, ≈40% of the landscape is water, featuring an abundance of water bodies, including lakes, ponds, and reservoirs (*22*). Hence, opportunities for exposure to natural water bodies are common during routine bathing, swimming, recreational, and occupational activities. Chlorination of such a large number of natural water bodies is neither feasible nor practical. Hence, prevention strategies need to focus on reducing high-risk exposures, particularly during monsoon and postmonsoon months. Risk communication should advise persons with skin lesions, ulcers, recent facial trauma, or recent nasal surgery to avoid exposure to natural water bodies.

Use of nonchlorinated water from household or public wells for domestic purposes was also associated with *Acanthamoeba* spp. AME and accounted for a substantial PAF. In our study, domestic water use referred to activities involving direct contact with the face, head, skin, or body, such as bathing or washing the face or hair, and did not refer to ingestion. Many households in Kerala depend on individual or public wells. Heavy rainfall, flooding, sewage contamination, organic matter, and algal growth might contribute to contamination or proliferation of FLAs in poorly maintained wells. Periodic chlorination and maintenance of wells, especially wells used for bathing, face washing, hair washing, and wound care, are practical interventions that can be promoted through local administrative systems.

*Acanthamoeba* infections have frequently been reported among immunocompromised persons (*19*), although studies from India have reported *Acanthamoeba* infection in persons without documented immunocompromising conditions (*23*–*28*). In our study, most rPCR-confirmed *Acanthamoeba* spp. case-patients did not have documented immunocompromising conditions in available records. Although immunocompromised status was associated with moderate increase in odds of illness in our analysis, the CIs were imprecise (MOR 1.9 [95% CI 0.8–4.7]). Therefore, the role of host susceptibility cannot be excluded. In addition, immune status might not have been completely ascertained, which could have resulted in exposure misclassification. Further studies are needed to better understand the host, pathogen, and environmental determinants of *Acanthamoeba* spp. AME in Kerala.

In our study, nasal rinsing and nasal ablution were not associated with rPCR-confirmed *Acanthamoeba* spp. illness. Previous studies have suggested that the use of nonsterile water may increase the risk for FLA infection (*29*). In Kerala, nasal ablution is widely practiced, especially among certain religious communities, so the high background prevalence of that exposure might have limited our ability to detect the association.

Lower socioeconomic status was associated with AME in univariable analysis and might influence behavioral exposures identified in our study. We included socioeconomic status in the directed acyclic graphs as a potential confounder because it could influence access to safe domestic water, reliance on nonchlorinated wells, and use of natural water bodies for bathing or other routine activities. We therefore adjusted socioeconomic status in the multivariable conditional logistic regression model. Risk communication strategies should prioritize socioeconomically vulnerable communities through improved access to safe water and routine maintenance and chlorination of wells.

A limitation of our study was including case-patients whose diagnosis was by wet-mount microscopy in our descriptive epidemiology. Wet-mount microscopy provides rapid laboratory evidence of motile trophozoites; however, it is less specific than rPCR and does not reliably identify the species of FLA. Although wet-mount examinations were performed independently by 2 trained microbiologists, some misclassification is possible. Second, we restricted the case–control study to rPCR-confirmed *Acanthamoeba* spp. cases from 4 districts, which might not represent all AME cases in Kerala or AME caused by other FLA. We did not compare rPCR-confirmed *Acanthamoeba* spp. case-patients with immunocompromised controls, and the immune status among case-patients may have been incompletely ascertained. Third, the analytical study had a small sample size, resulting in wide CIs for some exposures. Fourth, the incubation period for *Acanthamoeba* spp. GAE can range from days to several weeks (*13*). We used a 3-month referent exposure period in accordance with expert consultation; we did not capture any behavior or practices beyond the 3-month period. Nevertheless, we expected that study participants would perform routine behaviors such as swimming or bathing frequently, and a 3-month referent exposure might be sufficient to capture such activities. Fifth, after the surge of AME cases in the state, Kerala public health authorities issued regular messages discouraging swimming or bathing in natural water bodies and launched widespread campaigns to chlorinate wells (*16*). Those actions might have led case-patients or their relatives to recall behaviors related to swimming or bathing in natural water bodies more readily than controls, resulting in differential recall bias. We attempted to minimize that difference by using a standardized questionnaire and identical reference periods for cases and controls but could not exclude differential recall. Further studies combining pathogen-specific surveillance, molecular testing, environmental sampling, and immune-status assessment are needed to better understand *Acanthamoeba* spp. CNS infection in Kerala.

In conclusion, Kerala reported an increase in laboratory-supported AME cases in 2025. A*canthamoeba* spp. amebae accounted for most rPCR-confirmed infections. Cases increased during monsoon and postmonsoon months, and the overall case-fatality rate was 25.8%. In our analytical study, AME was associated with washing ulcers or skin lesions in natural water bodies, exposure to natural water bodies after maxillofacial injury or nasal surgery, use of nonchlorinated well water for domestic purposes, and exposure to natural water bodies and forceful entry of water into the nose. Public health interventions should focus on maintenance and chlorination of domestic wells and limiting exposure to natural water bodies for persons with wounds or recent facial or nasal procedures. 

Appendix Additional information from study of amebic meningoencephalitis, Kerala, India, 2025.
