# Supplementary material for: Epidemiology of and Risk Factors for Amebic Meningoencephalitis, Kerala, India, 2025
Source: Emerg Infect Dis. 2026 Sep;32(9):1402–12. doi: 10.3201/eid3209.260492 (PMC13555710; doi:10.3201/eid3209.260492)
Supplement: Supplementary file 1 [file 26-0492-Techapp-s1.pdf]

Article DOI: https://doi.org/10.3201/eid3209.260492

EID cannot ensure accessibility for supplementary materials supplied by authors. Readers who have difficulty accessing supplementary content should contact the authors for assistance.

Epidemiology of and Risk Factors for Amebic Meningoencephalitis, Kerala, India, 2025

Appendix

Laboratory Diagnosis

Molecular assays included conventional pan-genus FLA and genus-specific PCR for *Acanthamoeba*, *Naegleria*, and *Balamuthia* species and real-time PCR assays, the *Acanthamoeba*-specific primer pair used was the forward primer (5′-GGCCCAGATCGTTTACCGTGAA-3′) and the reverse primer (5′TCTCACAAGCTGCTAGGGAGTCA-3′) (*1*). The conventional PCR amplified products were sequenced for confirmation. The *Naeglaria*-specific primer pair used was the forward primer NaeglF192 (3′-GTG CTG AAA CCT AGC TAT TGT AAC TCA GT-5′), and the reverse primer (5′-CAC TAG AAA AAG CAA ACC TGA AAG G-3′) (*2*).

References

1. Sankar P, Moorkoth AP, Khurana S, Mohan A, Vadekkandyil S, M SA, et al. Microbiological profile of meningoencephalitis due to pathogenic free-living amoebae: a case series from Kerala, India. Cureus. 2026;18:e103922. PubMed https://doi.org/10.7759/cureus.103922

2. Qvarnstrom Y, Visvesvara GS, Sriram R, da Silva AJ. Multiplex real-time PCR assay for simultaneous detection of *Acanthamoeba* spp., *Balamuthia mandrillaris*, and *Naegleria fowleri*. J Clin Microbiol. 2006;44:3589–95. PubMed https://doi.org/10.1128/JCM.00875-06

Appendix Table. CSF profile of AME cases

Parameter (n = 128)	Category / Summary	Value
Total cell count (/µL)	Median (IQR)	12 (7–50)
	Range	0–12,900
	Missing, n	2
Protein (mg/dL)	Median (IQR)	67.6 (43–94)
	Range	0.5–5763
	Missing, n	1
Glucose (mg/dL)*	Median (IQR)	69.5 (59–91)
	Range	2–209
	Missing, n	2
Blood sugar/RBS (mg/dL)	Median (IQR)	132.5 (107–182)
	Range	71–850
	Missing, n	36
CSF: Blood glucose ratio	Median (IQR)	0.56 (0.40–0.68)
	Missing, n	37
Pleocytosis (n = 126)	Normal (≤5 cells/µL)	23 (18.3%)
	Mild (6–50 cells/µL)	73 (57.9%)
	Moderate (51–500 cells/µL)	16 (12.7%)
	Marked (>500 cells/µL)	14 (11.15)
Predominant cell type (n = 128)	Lymphocytic	90 (70.3%)
	Neutrophilic/Polymorph predominant	3 (2.3%)
	Mixed	3 (2.3%)
	Non-lymphocytic/Other	12 (9.4%)
	Unspecified/Unknown	20 (15.6%)
CSF: Blood glucose ratio (n = 91)	<0.5	34 (37.4%)

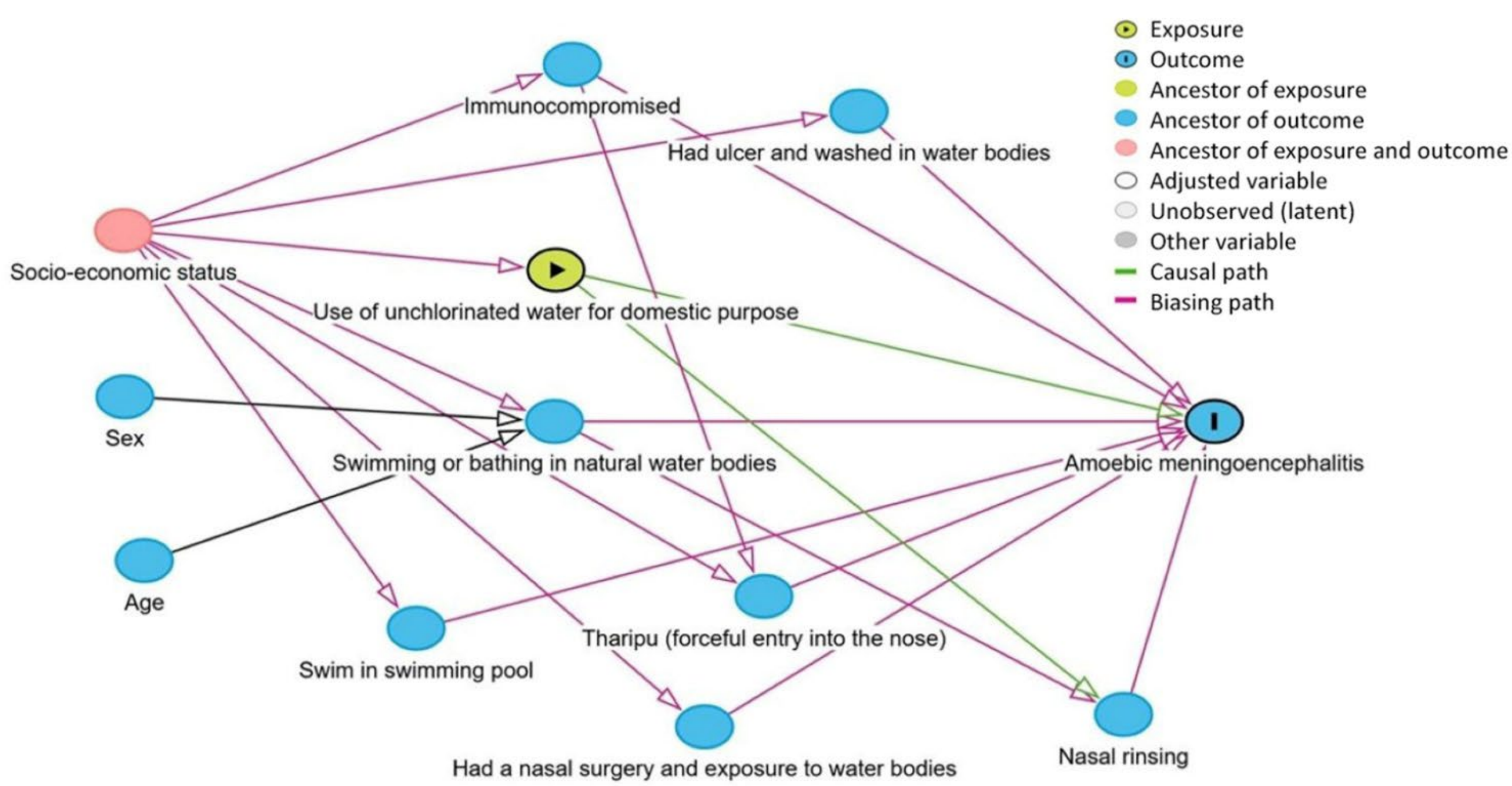

Appendix Figure 1. Directed acyclic graph of risk factors associated with AME, Kerala, 2025.

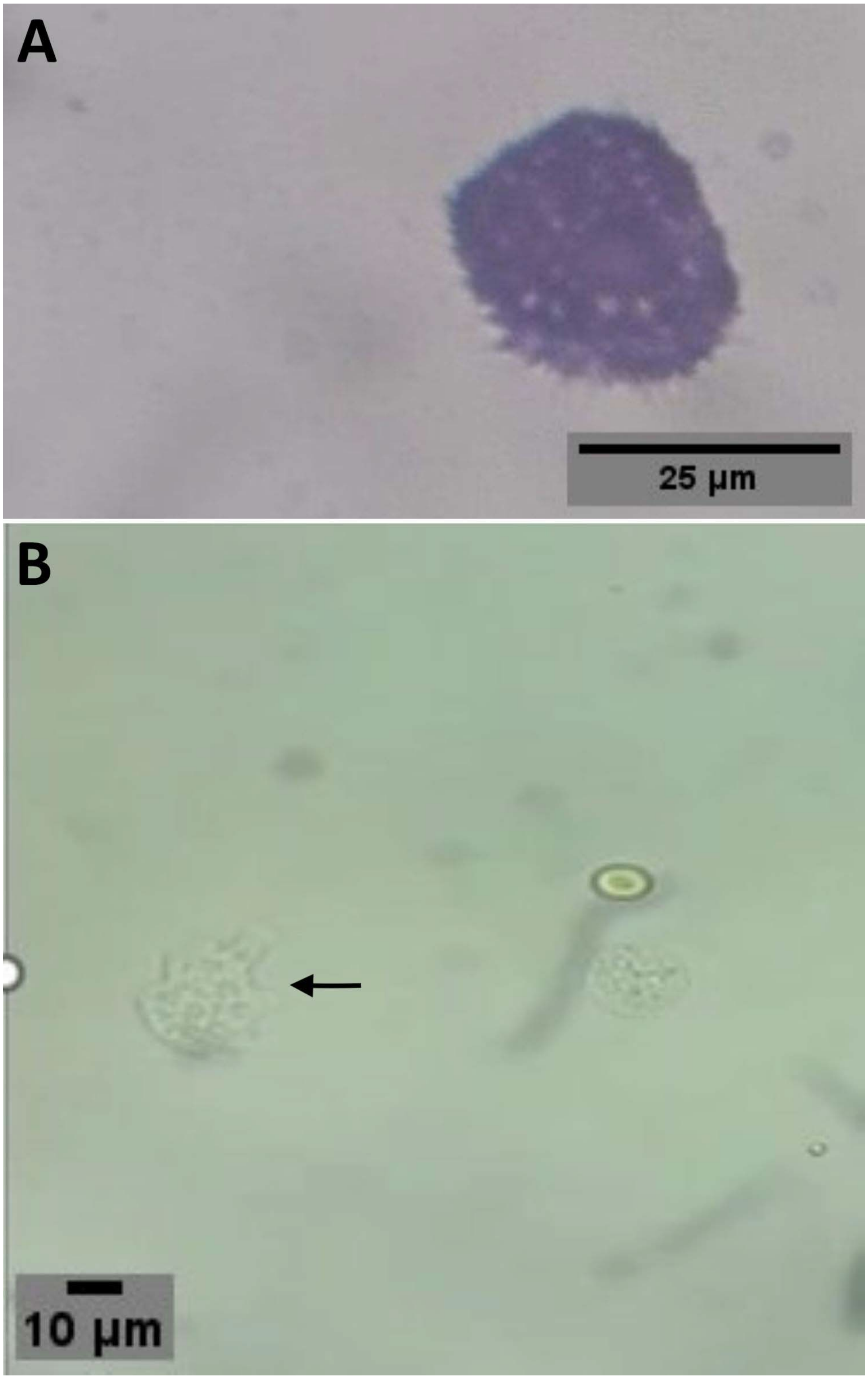

Appendix Figure 2. Microscopic images of free-living amoeba. A) *Acanthamoeba* trophozoite: Giemsa stain showing Acanthopodia, nucleus, and contractile vacuoles (under oil immersion, original magnification ×100). B) *Naegleria* trophozoite in CSF wet mount.
